# Eye Degeneration and Loss of *otx5b* Expression in the Cavefish *Sinocyclocheilus tileihornes*

**DOI:** 10.1007/s00239-019-09901-8

**Published:** 2019-07-22

**Authors:** Zushi Huang, Tom Titus, John H. Postlethwait, Fanwei Meng

**Affiliations:** 1 Institute of Zoology, Chinese Academy of Sciences, Beijing, 100101 China; 2 Institute of Neuroscience, University of Oregon, Eugene, OR 97403 USA

**Keywords:** Cavefish, *Sinocyclocheilus*, Eye, Retina, *otx5*, Phototransduction, *crx*

## Abstract

Cave animals possess remarkable phenotypes associated with existence in their dark environments. The Chinese cavefish *Sinocyclocheilus tileihornes* shows substantial eye degeneration, a trait shared by most cave species. The extent to which independent evolution of troglomorphic traits uses convergent molecular genetic mechanisms is as yet unknown. We performed transcriptome-wide gene expression profiling in *S. tileihornes* eyes and compared results with those from the closely related surface species *S. angustiporus* and an independently derived congeneric cavefish, *S. anophthalmus*. In total, 52.85 million 100 bp long paired-end clean reads were generated for *S. tileihornes*, and we identified differentially expressed genes between the three possible pairs of species. Functional analysis of genes differentially expressed between *S. tileihornes* and *S. angustiporus* revealed that phototransduction (KEGG id: dre04744) was the most significantly enriched pathway, indicating the obvious differences in response to captured photons between the cavefish *S. tileihornes* and the surface species *S. angustiporus*. Analysis of key genes regulating eye development showed complete absence of *otx5b* (*orthodenticle homolog 5*) expression in *S. tileihornes* eyes, probably related to degradation of rods, but normal expression of *crx* (*cone*-*rod homeobox*). The enriched pathways and Otx5 are involved in phototransduction, photoreceptor formation, and regulation of photoreceptor-related gene expression. Unlike the *S. tileihornes* reported here, *S. anophthalmu*s has reduced *crx* and *otx5* expression. These results show that different species of cavefish within the same genus that independently evolved troglodyte characteristics can have different genetic mechanisms of eye degeneration.

**Electronic supplementary material:**

The online version of this article (10.1007/s00239-019-09901-8) contains supplementary material, which is available to authorized users.

## Introduction

Blind cavefish provide an excellent model for the study of the genetic mechanisms for the evolution of developmental change. These remarkable fish have evolved in constant darkness over years and developed a series of compelling adaptive features (Borowsky 2018; Protas et al. 2008). Different species of cave-dwelling fish have evolved a series of adaptations that scale with the antiquity of independent evolution in the dark and the proximity of their habitat to the mouth of the cave. Whereas the eyes of some cave-dwelling fish species are merely reduced in size, others are small and internal and appear to be entirely blind. Thus, cavefish rely mostly on non-visual organs to sense their environment, find food and mates, and avoid predation (Borowsky 2018; Jeffery 2009; Protas et al. 2007; Stemmer et al. 2015). Nearly 200 species of cavefish have been found around the world. Of these, the Mexican blind tetra *Astyanax mexicanus* has the best-studied cave populations, and the *Sinocyclocheilus* genus contains the most reported cave species. The heads of cave-dwelling *Sinocyclocheilus* species exhibit surprising diversity in morphology, and their eyes show a broad range of phenotypes, from small reductions in eye size to almost complete loss (Borowsky 2018; Meng et al. 2018; Zhao et al. 2011).

The vertebrate eye develops under the precise control of a regulatory network that includes genes encoding numerous transcriptional regulators, such as orthodenticle homolog-2 (Otx2), cone-rod homeobox (Crx), SRY-box 2 (Sox2), paired box 6 (Pax6), and diffusible cell signals, including sonic hedgehog (Shh) (Gregory-Evans et al. 2013; Hennig et al. 2008; Matsushima et al. 2011; Swaroop et al. 2010). In *A. mexicanus*, degeneration of optic tissues probably resulted from apoptosis in the lens, which is induced by the down-regulation of *sox2,* or the expansion of *shh* expression in the cavefish (Ma et al. 2014; Pottin et al. 2011; Yamamoto et al. 2004). In contrast, the reduction of eye size and function in *S. anophthalmus* (*Sano*) appears to be due to a lens-independent mechanism related to reduced proliferation in ciliary marginal zone and the down-regulation of *crx* (Meng et al. 2013a). Transcriptome sequencing of embryonic and adult cavefish of *A. mexicanus* has shown that the number of eye-related genes with mutations increased significantly over evolutionary time (Hinaux et al. 2013; Stahl and Gross 2017). Mutations in these genes, such as *FKBP prolyl isomerase 3*, *enolase 3*, *calcyphosine*-*like a*, *ribosomal protein L13*, may be explained by relaxed selection in the dark environment and may have played an important role in the degeneration of cavefish eyes (Hinaux et al. 2013). The proliferation of retinal cells is reduced in the cavefish *S. anophthalmus*, and transcriptome analysis showed that the degenerated eyes in *S. anophthalmus* have strongly down-regulated expression of *crx* (Meng et al. 2013a). The expression of *otx2,* however, which directly regulates *crx* transcription (Nishida et al. 2003), is known to not change significantly in the eye of *S. anophthalmus* (Meng et al. 2013a). There may, therefore, be some changes in the genes involved in the regulation of *crx* expression that requires further study. Although previous work has identified genes—such as *crx*, *otx2*, *shh*, *sox2*, and *pax6*—associated with developmental defects in the eyes of cave *A. mexicanus* and *Sinocyclocheilus*, no inactivating mutations have been found in the coding portions of these genes (McGaugh et al. 2014; Meng et al. 2013a). In addition to the thin retinas and more sparsely populated photoreceptors found in cavefish *S. anophthalmus* and *S. tileihornes* (*Stil*) relative to that of surface species *S. angustiporus* (*Sang*), we also found that the arrangement of rods in cavefish *S. tileihornes* (*Stil*) was disorganized (Meng et al. 2013b). Whether eyes degenerate in different cavefish species of the same genus as a result of mutations in the same genes, mutations in distinct genes in the same pathway, or mutations in genes in different regulatory pathways remains an unsolved problem.

To help address this question, we used RNA-seq to perform transcriptome sequencing for examination of gene expression levels in the eyes of three *Sinocyclocheilus* species. First, we identified differences in eye transcriptomes of *S. tileihornes* compared with that of the surface species *S. angustiporus* and another cavefish, *S. anophthalmus. Sinocyclocheilus angustiporus* is more closely related to *S. anophthalmus* than *S. tileihornes* see cladogram in Fig. S1 (Zhao and Zhang 2009). Next, we analyzed differentially expressed genes using gene ontology (GO) and pathway functional enrichment analysis. We found that phototransduction was the most significantly enriched pathway between cavefish *S. tileihornes* and the surface species *S. angustiporus*. While expression of *crx* and *otx5* is depressed in *S. anophthalmus* eyes (Meng et al. 2013a), results reported here showed that *crx* expression was normal and *otx5* expression was down-regulated in *S. tileihornes,* indicating different mechanisms of eye reduction in the two cave species within same genus. Despite their names, the mammalian gene unfortunately called *Crx* is the ortholog of the gene called *Otx5* in all other vertebrates (Plouhinec et al. 2003). Zebrafish has two co-orthologs of the human *CRX* gene (Plouhinec et al. 2003) that derive from the TGD event (Catchen et al. 2009) and these genes are called, inappropriately, *crx* (ZFIN ID: ZDB-GENE-010403-1) and *otx5*. (ZFIN ID: ZDB-GENE-030508-1), which obscures the fact that they are ohnologs from the teleost TGD (Fig. S2). Common carp and goldfish (cypriniformes, cyprinidae, cyprininae) experienced a genome duplication event about 8 million years ago (Meng et al. 2013a; Wang et al. 2012) long after the cyprininae lineage diverged from the zebrafish lineage (cypriniformes, cyprinidae, danioninae), and this event is shared by *Sinocyclocheilus* (Meng et al. 2013a). As a consequence of the carp genome duplication (CaGD), at least some *Sinocyclocheilus* species have two co-orthologs of the zebrafish *otx5* gene derived from the CaGD, currently called *otx5a* and *otx5b*. We verified the expression levels of *otx5a* and *otx5b* by qRT-PCR and found that expression of *otx5b* was completely lost in *S. tileihornes*. This study provides a valuable resource to further elucidate the molecular mechanisms behind degradation in cavefish eyes and shows that even species within the same genus can have different mechanisms of eye degeneration.

## Materials and Methods

### Animal Samples and RNA Extraction

**Fig. 1:**
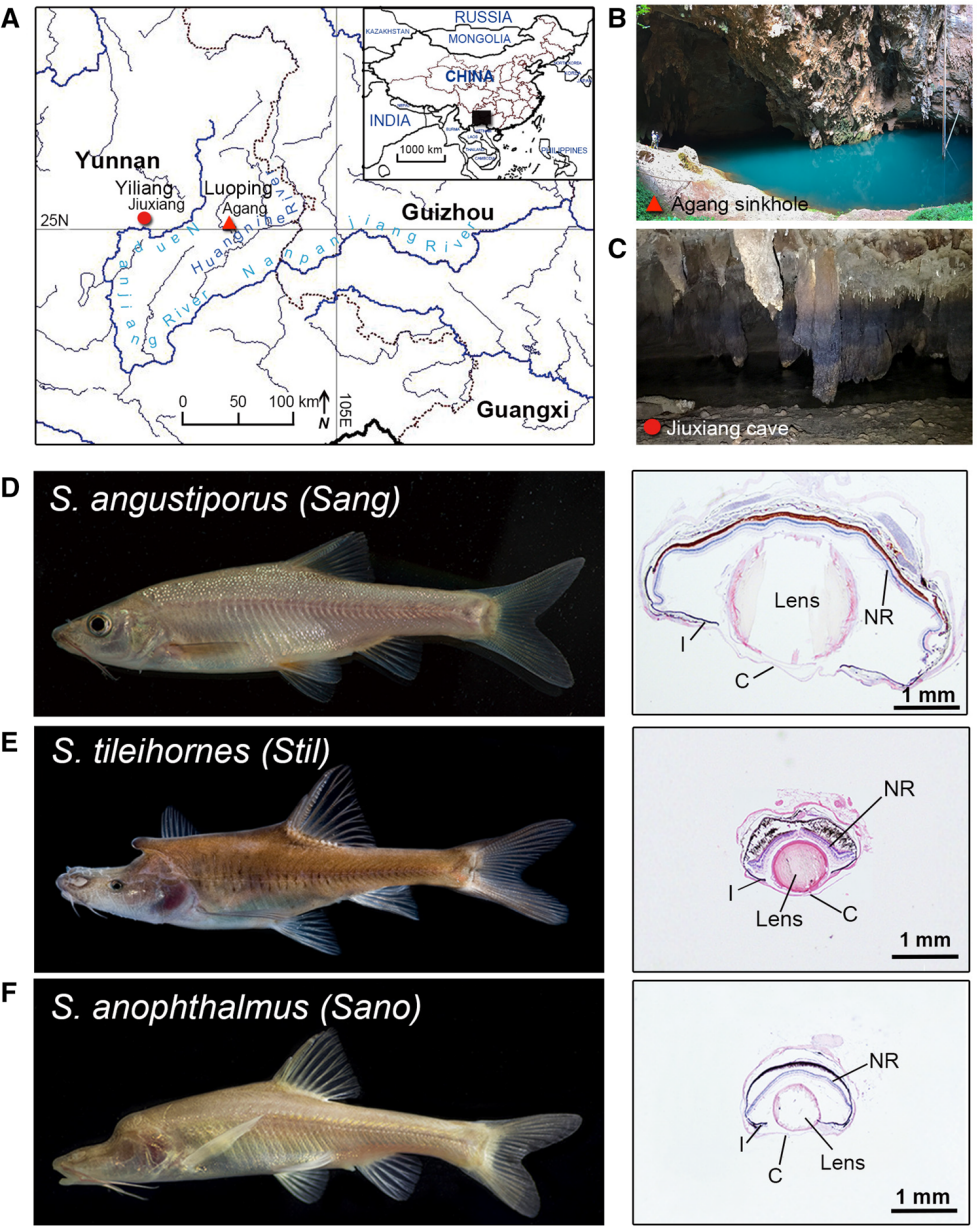
**a** Collection sites for *Sinocyclocheilus*. The inset is a map of China with a filled rectangle representing the area of the larger map. The red triangle indicates collection locations of the cavefish *S. tileihornes* (*Stil*) and surface fish *S. angustiporus* (*Sang*) (Agang sinkhole, N 25.00905°, E 103.59256°). The red circle indicates the collection location of the cavefish *S. anophthalmus* (*Sano*) (N 25.05478°, E 103.37975°). Longitude and latitude: 105E and 25N. **b** The collection site of *S. tileihornes* and *S. angustiporus.***c** View into the Jiuxiang cave. **d** Surface fish *Sang* and H&E stained sections of its eye. **e** Cavefish *Stil* and H&E stained sections of its eye. **f** Cavefish *Sano* and H&E stained sections of its eye. *C* cornea, *I* iris, *NR* neural retina. Scale bar in **d**–**f** 1 mm

Adult *Sinocyclocheilus* fish were collected in Yunnan province, China (Fig. 1a). *Sinocyclocheilus tileihornes* can be collected only in a sinkhole connected to the Huangnihe River in Agang Town, Luoping County. The fish are suspected to live in the underground river connected to the sinkhole, swimming out only occasionally. Because escape from the cave is extremely rare, only eight *S. tileihornes* individuals were captured by our group over several collection attempts. *Sinocyclocheilus angustiporus*, a normally sighted *Sinocyclocheilus* species, was collected living at the surface of the Huangnihe River sinkhole (Fig. 1a, b). Another cave species, *S. anophthalmus*, was collected from Jiuxiang Cave, Yiliang County (Fig. 1a, c).

Fish were generally euthanized as soon as possible after fish collection in the field, and their eyes dissected and placed into RNAlater (Ambion, Austin, TX, USA). The remaining fish were brought back to the laboratory where they were maintained in aquaria. *Sinocyclocheilus tileihornes* and *S. anophthalmus* were maintained in a dark environment in the laboratory, and *S. angustiporus* specimens were exposed to a photoperiod of 3-h light/21-h dark, which imitates their conditions in the wild due to shading by the walls of the sinkhole. Total RNA from the left eyes of the three species (*n *= 3 for each species) was isolated using TRIzol reagent (Invitrogen, Carlsbad, CA, USA), and the right eyes were fixed for histological analysis.

### Hematoxylin and Eosin (H&E) Staining, In Situ Hybridization (ISH), and Clone Sequencing

Before cryosectioning, the eyes of cave and surface fish were stored in 30% sucrose at 4 °C overnight. The sections were stained with hematoxylin and eosin (H&E). For ISH, RNA probes were generated using Roche digoxygenin from *Sinocyclocheilus* cDNAs. The following primers were used to clone *otx5* probes in PCR reactions: *otx5*-*F*: 5′-TGTGGTTTAAGAACCGTCGTG-3′ and *otx5*-*R*: 5′-GAACTTCCAGGAGTTCTGGTC-3′, which amplifies the exon3 of *otx5*. PCR amplification products were recovered from the gels with a DNA purification kit according to the manufacturer’s instructions (DP209; Tiangen Biotech) and then cloned into the pGM-T vector (VT202; Tiangen Biotech) in *E. coli DH5a*. Six clones were sequenced for every sample using T7 and Sp6 universal sequencing primers. Sequence-verified clones were used to generate antisense probes using SP6/T7 enzymes. ISH was performed with the color visualized using NBT/BCIP as described previously (Meng et al. 2013b).

### Illumina Sequencing, Assembly, and Identification of Differential Gene Expression in Surface Fish and Cavefish Eyes

After oligo(dT) selection with MicroPoly(A)Purist (Ambion, Austin, TX, USA) according to the manufacturer’s protocol, the whole transcriptome library of the eyes of two *S. tileihornes* individuals was constructed following established protocols (Meng et al. 2013a). One sample of 200–400 bp insert length was sequenced on a HiSeq 2000 instrument (Illumina, San Diego, CA, USA) using paired-end 100-nucleotide reads. Sequencing data were submitted to the NCBI Sequence Read Archive. Reads were cleaned by removing adaptor sequences and low-quality reads containing > 5% ambiguous bases (N) and < 50% bases with quality > 19 using fqtools (Droop 2016). We used Trinity 2.1.1 (Grabherr et al. 2011) to generate three *Sinocyclocheilus* transcriptome assemblies, one for each species (*S. tileihornes*, *S. angustiporus*, and *S. anophthalmus*) using default settings with 300 nt minimum contig length. Cap3 (Huang and Madan 1999) was used to generate a non-redundant *Sinocyclocheilus* transcriptome by merging contigs from the three species. We compared the combined *Sinocyclocheilus* transcriptome with the gene records of zebrafish (GRCz11) using Blastn with cut-off *E* value of 10^−3^. Bowtie 2 software (2.3.5) (Langmead et al. 2009) was then used to map reads to the non-redundant *Sinocyclocheilus* transcriptome with default settings. Reads that mapped onto *Sinocyclocheilus* transcriptome contigs that matched zebrafish gene models (GRCz11) were converted to FPKM values (fragments per kilobase of gene per million mapped fragments). During the identification of differentially expressed genes and subsequent GO analysis, the two paralogs from the CaGD were collapsed into one annotation unit corresponding to the zebrafish ortholog. Genes with a FPKM value of > 5 in at least one of the three species were used to run differential expression analysis using edgeR (Robinson et al. 2010). When comparing surface species to cave species, genes with a fold change (FC) > 2 and *P* < 0.05 were identified as up-regulated, and those with a FC < 0.5 and *P* < 0.05 were identified as down-regulated.

### Gene Ontology and Pathway Enrichment Analysis

GO and KEGG pathway enrichment analyses were performed on differentially expressed genes using KOBAS 3.0 (Xie et al. 2011). The results of the GO functional enrichment analysis were classified into three categories: biological process, cellular component, and molecular function. The formula we used to calculate the enrichment factor is that differentially expressed genes mapped to the particular pathway/number of background genes of this pathway. The GO categories for the differentially expressed genes with *P* values < 0.05 were regarded as significant compared with the background genes within this GO term. By using WEGO 2.0 (Ye et al. 2018), we created histograms with the GO classification of the differentially expressed genes. KEGG pathways with *P* values < 0.05 were considered to be enriched.

### Quantitative Real-Time Polymerase Chain Reaction (qRT-PCR)

We reconstructed phylogenetic topologies for *Sinocyclocheilus* species using the *crx* and *otx5* sequences from each species by maximum-likelihood (ML) and Bayesian inference (BI) methods (see Supplementary material). The CaGD paralogs of *crx* and *otx5* have several identifying nucleotide variations. Amplification primers were designed based on the sequence alignment of the transcriptomes and genomes of six *Sinocyclocheilus* species: *S. angustiporus*, *S. anophthalmus*, *S. anshuiensis*, *S. grahami*, *S. rhinocerous,* and *S. tileihornes* (Meng et al. 2013a; Yang et al. 2016). The primers could distinguish the expression levels of the *crxa*, *crxb*, *otx5a*, and *otx5b* in *Sinocyclocheilus*.

cDNA samples were constructed from total eye RNAs using a First-Strand cDNA Synthesis Kit (Invitrogen, Carlsbad, CA, USA). Real-time PCR was conducted with a CFX96 Real-Time PCR detection system (Bio-Rad, Singapore) and SYBR Green (TaKaRa, Dalian, China) chemistry. Primer sequences were as follows (5′-3′): *β*-*actin*-*F*: GAAGATCAAGATCATTGCTCCC and *β*-*actin*-*R*: ATGTCATCTTGTTCGAGAGGT; *crxa*-*F*: TCGGGAGCGCACTACCTTC, *crxb*-*F*: TCGGGAGCGCACTACTTTT and *crxab*-*R*: CGGCATTTAGCACGACGGT; *otx5ab*-*F*: GCCTCCTCGTCCTACTTCAC, *otx5a*-*R*: AGCTTCCAGGACGCCGTTT and *otx5b*-*R*: AGCTTCCAGGCCGTTTGGT. We used *β*-*actin* as a reference gene. The relative expression levels of *crx* and *otx5* were normalized to the expression of the internal reference gene, *β*-*actin,* using the relative Ct method. Three biological replicates were used for each gene. Statistical analysis of the data was performed using a two-tailed Student’s *t* test using Microsoft Excel. *P < 0.01.

## Results and Discussion

Our previous studies analyzed eye morphology and the expression pattern of *rhodopsin* (*rho*) by in situ hybridization in the cavefish *S. tileihornes* (Meng et al. 2013b), but we had not conducted a study at the transcriptome level to identify differentially expressed genes. Cavefish *S. tileihornes* and *S. anophthalmus* have different degrees of eye defects relative to surface fish *S. angustiporus*. However, histological sections showed that adults of both of these two cave species have what appear to be histologically normal, but small lenses, in contrast to *A. mexicanus* whose lens disappears (Fig. 1d–f). Here, we compared transcriptomic differences in two cavefish species and one closely related surface species to provide genome-wide insights into the genetic changes that accompanied adaptation to dark environments.

### RNA-Seq and Identification of Differentially Expressed Genes

**Fig. 2:**
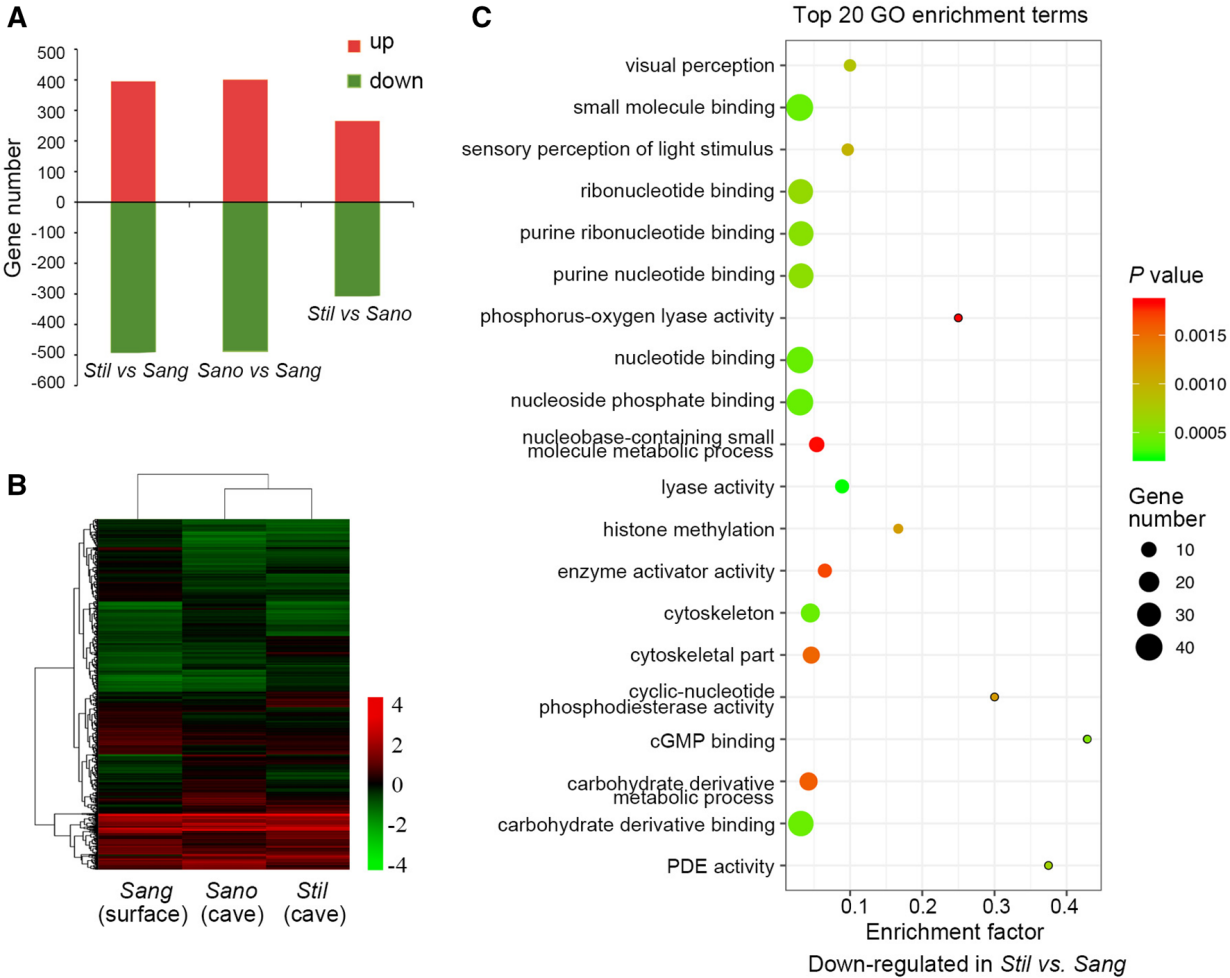
Identification and analysis of differentially expressed genes in *Sinocyclocheilus* species. **a** The number of up-regulated and down-regulated differentially expressed genes from comparisons among three species. **b** Hierarchical clustering and heat map of differentially expressed genes in the three species. Each column represents a species, and each row represents a gene. Green indicates the low level of gene expression, and red means high level. **c** Significant enrichment of down-regulated differentially expressed genes between *S. tileihornes* and *S. angustiporus* in the top 20 GO terms selected by the *P* values. The x-axis represents the enrichment factor; the y-axis represents the GO terms. Point size indicates the number of genes enriched in the GO term. Colour means the *P* value

The transcriptome of *S. tileihornes* eyes was analyzed by RNA-Seq. We generated 52.85 million 100 bp long paired-end reads for *S. tileihornes*. The *S. tileihornes* de novo transcriptome assembly contained 152,512 contigs composed of 90,898,926 bases. We constructed a combined *Sinocyclocheilus* transcriptome from the new *S. tileihornes* assembly plus the previous transcriptome assemblies of *S. anophthalmus* and *S. angustiporus* that contained 59,631 contigs with N50 of 1318, of which 41,134 contigs matched 15,649 zebrafish genes (GRCz11) (Table S1). 36.98 million (61.65%) *S. angustiporus* reads, 26.68 million (57.33%) *S. anophthalmus* read and 30.06 million (56.88%) *S. tileihornes* reads mapped to the combined *Sinocyclocheilus* reference transcriptome using Bowtie 2 software. Supplementary Table S2 lists genes according to their change in expression level fold change (FC). Among 9958 unique genes with FPKM values > 5 in at least one of the three *Sinocyclocheilus* eye transcriptomes, we identified 891 differentially expressed genes (395 up and 496 down) in *S. tileihornes* cavefish compared with *S. angustiporus* surface fish, 889 differentially expressed genes (401 up and 488 down) in *S. anophthalmus* cavefish compared with *S. angustiporus* surface fish, and 605 differentially expressed genes (266 up and 339 down) in *S. tileihornes* cavefish compared with *S. anophthalmus* cavefish (Fig. 2a). The total number of differentially expressed genes identified from the three comparisons was 1560.

Hierarchical clustering displayed as a heat map helped to determine the profiles of the 1560 differentially expressed genes (Fig. 2b). Results showed that the gene expression levels of cavefish *S. tileihornes* and *S. anophthalmus* exhibit similar clustering and expression patterns; however, *S. anophthalmus* and *S. angustiporus* are more closely related than either are with *S. tileihornes,* according to the phylogenetic relationships based on the *cytochrome b* gene, *crx* and *otx5* genes (Figs. S1 and S3) (Xiao et al. 2005; Zhao and Zhang 2009). We also found that 245 and 135 differentially expressed genes were co-down-regulated or co-up-regulated, respectively, in both cavefishes compared with surface species (Table S2, sheets 2–3). Three pathways were enriched (*P* < 0.05) in the co-down-regulated gene group (Table S3). The most significantly enriched pathway was phototransduction (KEGG ID: dre04744) (*P* = 0.0003 and FDR = 0.0129). This might be related to the similar phenotype of eye degradation between cavefish *S. tileihornes* and *S. anophthalmus* (Meng et al. 2013a; Meng et al. 2013b). These results indicate that gene expression in the eyes of both independent cave lineages has evolved a similar profile during the adaptation of these animals to cave environments.

### GO and Pathway Enrichment Analysis of the Differentially Expressed Genes

**Fig. 3:**
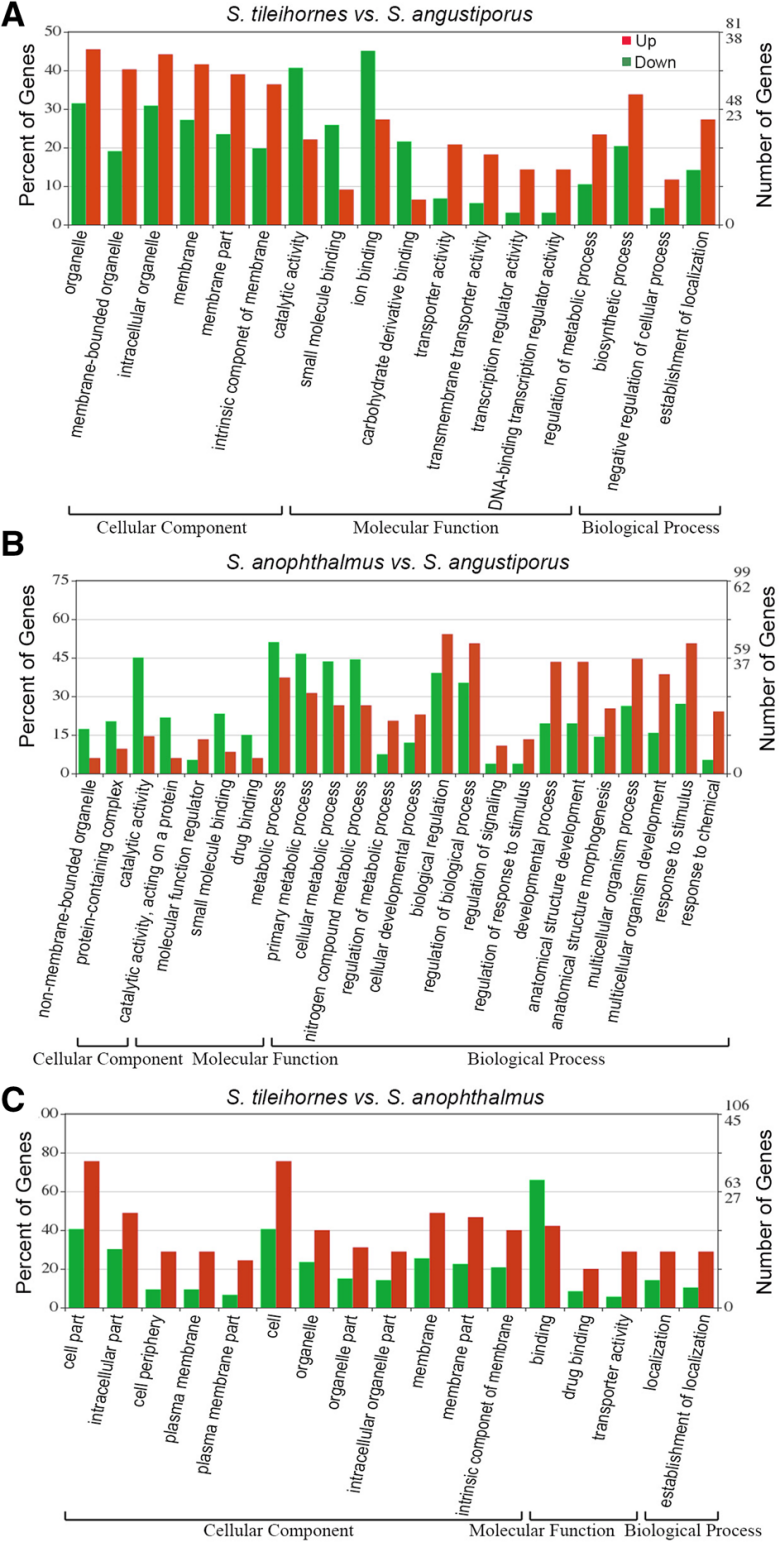
GO classifications of differentially expressed genes from three comparisons. **a** Comparison of GO classification of the differentially expressed genes in *S. tileihornes* versus *S. angustiporus*. **b** Comparison of GO classification of the differentially expressed genes in *S. anophthalmus* versus *S. angustiporu*. **c** Comparison of GO classification of the differentially expressed genes in *S. tileihornes* versus *S. anophthalmus*. Level-3 GO terms with significant gene number differences are summarized in three categories: cellular component, biological processed, and molecular function. The x-axis represents the category, the y-axis represents the percentage and number of differentially expressed genes. Green bars are down-regulated genes, and red bars are up-regulated genes

GO functional enrichment analysis was based on the differentially expressed genes from three separate comparisons: *Stil* versus *Sang*, *Sano* versus *Sang* and *Stil* versus *Sano*, separately. The significant level-3 GO terms in the three categories are shown in Fig. 3. The enrichment analysis showed that GO terms related to the visual perception (*P* = 0.0008), sensory perception of light stimulus (*P* = 0.0010), 3′,5′-cyclic-nucleotide phosphodiesterase (PDE) activity (*P* = 0.0007), and cGMP binding (*P* = 0.0005) were significantly enriched in the down-regulated genes of *S. tileihornes* cavefish versus *S. angustiporus* surface fish (Fig. 2c). The GO enrichment analysis makes sense in terms of these histological findings. These enriched GO terms are all related to phototransduction. Previous studies have shown that the expression level and distribution of *rho* in the *S. tileihornes* retina were significantly reduced and its eyes do not respond to light (Meng et al. 2013b).

**Fig. 4:**
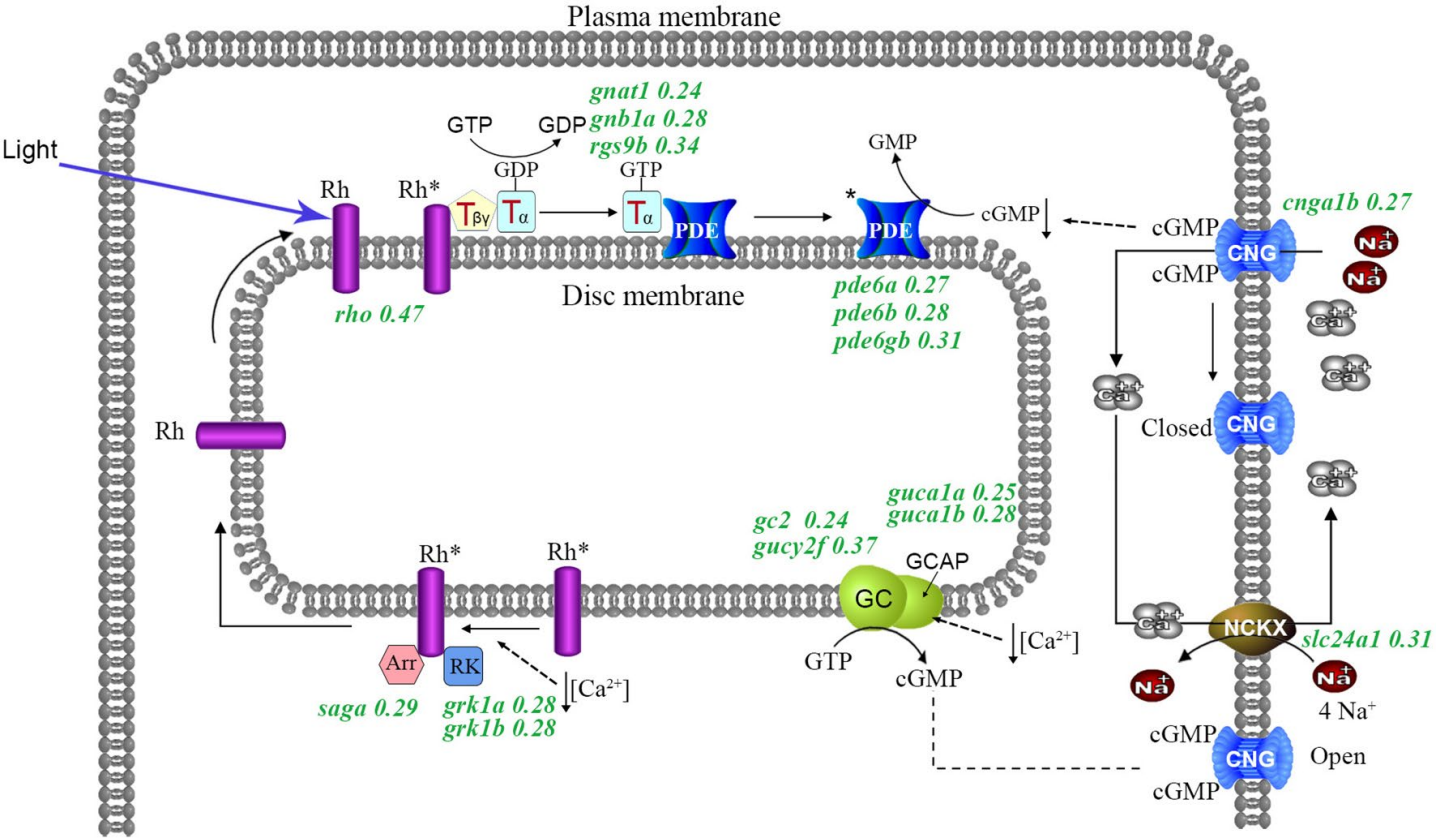
Scheme of the vertebrate phototransduction process redrawn and adapted from (Fain et al. 2010; Shichida and Matsuyama 2009). Numbers following genes indicate the fold changes of gene expression for differentially expressed genes in *S. tileihornes versus S. angustiporus*. *Rh* rhodopsin, Rh* activated form of rhodopsin, *GTP* guanosine triphosphate, *GDP* guanosine diphosphate, *T* transducin, *PDE* guanosine nucleotide phosphodiesterase, *cGMP* guanosine 3′,5′ -cyclic monophosphate, *GC* guanylate cyclase, *GCAP* guanylate cyclase activating protein, *GMP* guanosine monophosphate, *RK* rhodopsin kinase, *Arr* arrestin, *CNG* cGMP-gated channel), *NCKX* Na^+^/Ca^2+^-K^+^ exchanger

We identified four enriched pathways (*P* < 0.05) in the down-regulated gene group and five enriched pathways (*P* < 0.05) in the up-regulated gene group between *Stil* versus *Sang* (Table S3). The most significantly enriched pathway is “phototransduction”, by which photoreceptors convert light energy (photons) to electrical signals (Fain et al. 2010; Pepe 2001). In the cavefish *S. tileihornes* eye, 15 genes of a total of 34 background genes in the “phototransduction” pathway were down-regulated (Table S3 and Fig. 4). These 15 genes belong to different processes of phototransduction. Such as, following photon absorption, activated rhodopsin (*rho*, fold change, FC, is 0.47) catalyzes replacement of GDP by GTP on transducins, including *guanine nucleotide binding protein (G protein), alpha transducing activity polypeptide 1* (*gnat1*), FC = 0.24, *guanine nucleotide binding protein (G protein), beta polypeptide 1a* (*gnb1a*), 0.28, *regulator of Gprotein signaling 9 binding protein* (*rgs9b*), 0.34. Transducin-GTP activates the cGMP phosphodiesterase (PDE) (*pde6a* 0.27, *pde6b* 0.28, *pde6gb* 0.31), which in turn catalyzes the hydrolysis of cGMP. The reduced cGMP concentration leads to the closure of cGMP-gated channels (*cyclic nucleotide gated channel alpha 1b*, *cnga1b* 0.27) and the blockage of influx of Na^+^ and Ca^2+^. Next, the efflux of Ca^2+^ through Na^+^/Ca^2+^-K^+^ exchanger (*solute carrier family 24, member 1*, *slc24a1* 0.31) reduces the concentration of Ca^2+^ in cytoplasmic space, which in turn activates guanylyl cyclase activator protein (GCAP) (*guca1a* 0.25; *guca1b* 0.28) and guanylyl cyclase (GC, *gc2* 0.24; *gucy2f* 0.37), the level of cGMP rises and opens cGMP-gated channels. Rhodopsin kinase (*G protein*-*coupled receptor kinase 1 a*, *grk1a* 0.28, *grk1b* 0.28) and Arrestin (*S*-*antigen; retina and pineal gland (arrestin) a*, *saga* 0.29) inactivates *rhodopsin*, which becomes ready for another phototransduction cycle (Fig. 4). Down-regulation of these genes reflects the degenerated retina in *S. tileihornes* and likely contributors to the blindness in cavefish *S. tileihornes*. Among the differentially expressed genes between *S. tileihornes* and *S. angustiporus,* we also found two significant pathways, “Purine metabolism” (dre00230) and “Fructose and mannose metabolism” (dre00051) (Table S3). This result suggests that reduced expression of these metabolic-related genes might help to save energy and material expenditure in the eyes of *Sinocyclocheilus* cavefish, compared with the surface fish.

### Complete Loss of *otx5b* Expression

**Fig. 5:**
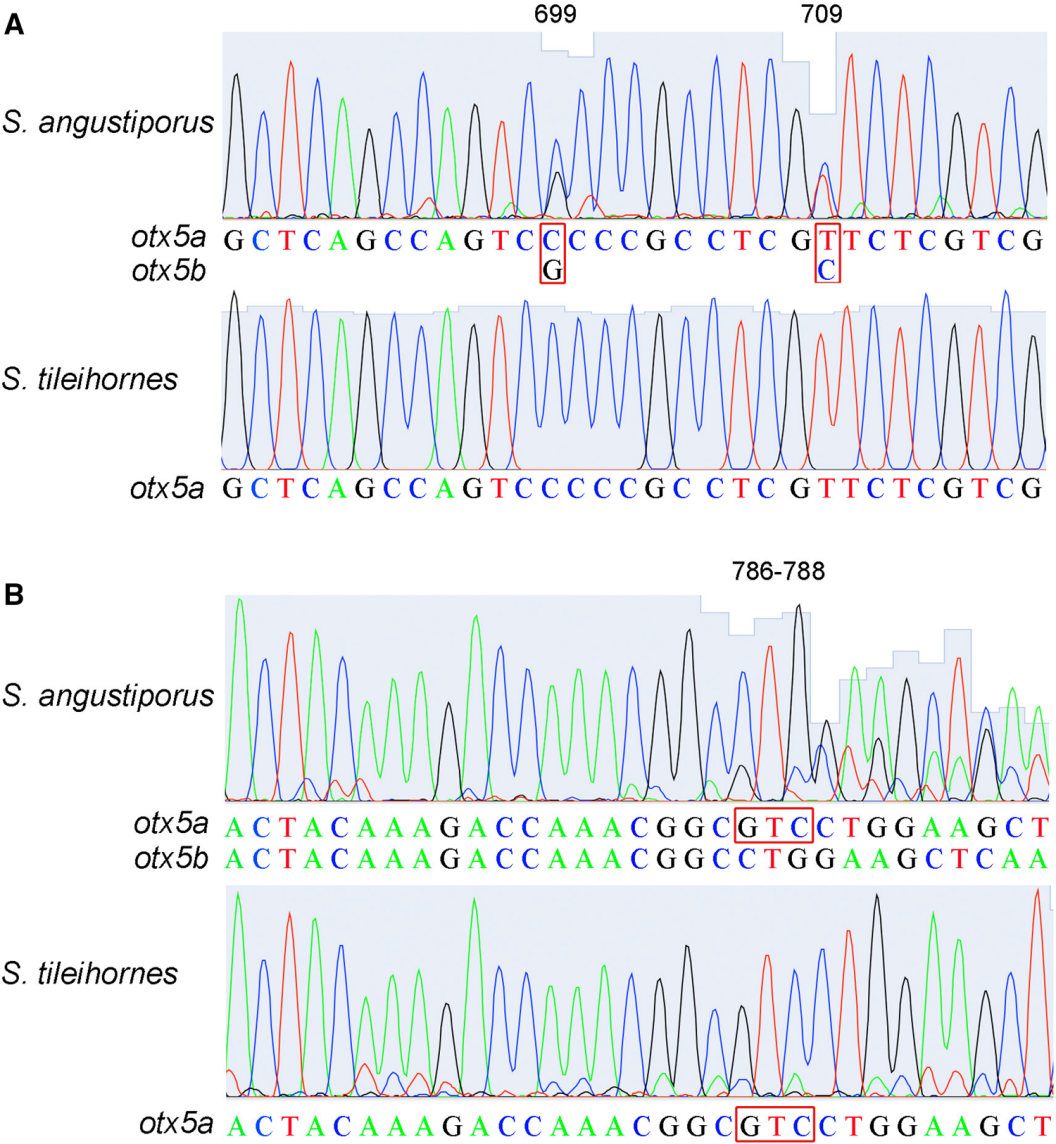
Sequence analyses of *otx5* in the cDNAs of *S. angustiporus* and *S. tileihornes* reveal the loss of *otx5b* in the cavefish *S. tileihornes*.** a** Sequencing results clearly show two single nucleotide polymorphisms (SNPs) sites with double peaks at position 699 and 709 between the CaGD ohnologs *otx5a* and *otx5b* in *S. angustiporus*. In contrast, *S. tileihornes* only has single peaks. **b** A 3-bp deletion at position 786–788 was discovered in *otx5b* of *S. angustiporus* and *S. anophthalmus* (Fig. S5a). The *otx5b* with 3-bp deletion was missing in *S. tileihornes*

**Fig. 6:**
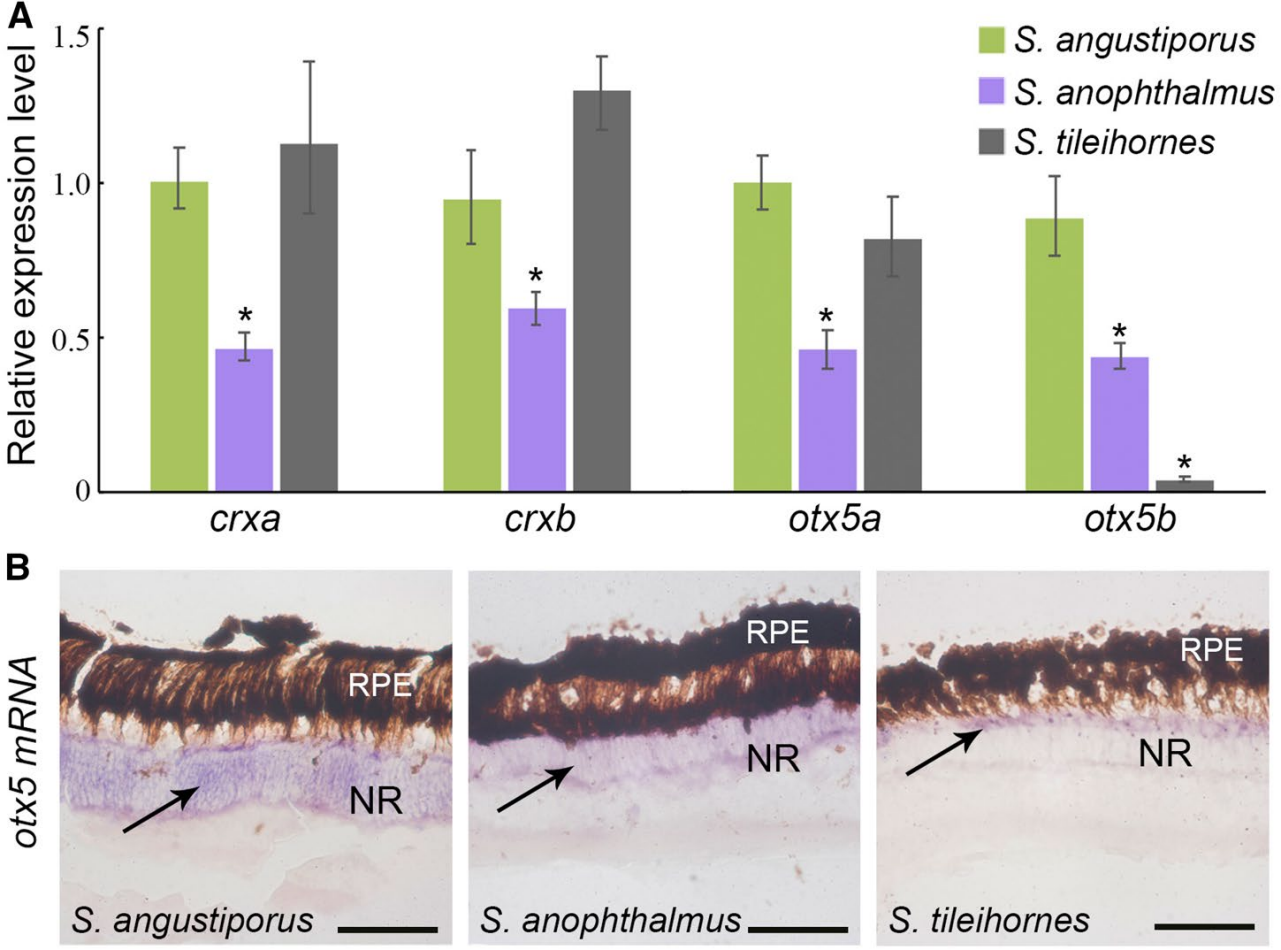
**a** To distinguish the expression level of different *crx* and *otx5* paralogs, we designed primers to detect *crxa*, *crxb*, *otx5a* and *otx5b* with qRT-PCR using RNA isolated from the eyes of each species. Expression levels of *crx* and *otx5* paralogs were quantified and normalized to* β*-*actin*. Relative expression values are the means of at least three independent experiments. Statistical analysis of the data was performed using a two-tailed Student’s *t* test using Microsoft Excel. *P < 0.01. **b** In situ hybridization was performed using probes for *otx5* on retinal sections from three species. The purple regions indicate the expression of *otx5* (black arrow). *NR* neural retina, *RPE* retina pigment epithelium. Scale bar: 50 μm

A whole genome duplication event, the teleost genome duplication (TGD), occurred at the base of the teleost radiation (Amores et al. 1998; Postlethwait et al. 1998) that produced two *otx5* ohnologs: *otx5* and *crx* (Plouhinec et al. 2003). In a previous study, we found that the expression of *otx5*, *crx,* and the genes regulated by *crx* were significantly down-regulated in the eye of the cavefish *S. anophthalmus* (Meng et al. 2013a). In *S. tileihornes*, we found here that the expression of *crx* was unchanged when compared with surface fish but that the expression of *otx5* and several phototransduction genes was still down-regulated, including *gnat1*, *gnat2*, *guca1a* and *guca1b*. We found no reduction in cone opsins. The expression of *rho* (rod opsin), however, decreased (FC, 0.47) (Table S2). This change in gene expression is consistent with our previous morphological results (Meng et al. 2013b), which showed that the main defect in the eye of *S. tileihornes* was related to rod cells. *Sinocyclocheilus* has two co-orthologs of the zebrafish gene *otx5* that we call *otx5a* and *otx5b* and are derived from the carp genome duplication (CaGD) (Meng et al. 2013a) (Fig. S3). When we visualized the sequence alignment of several down-regulated genes (*crx*, *gnat1*, *gnat2*, *nrl*, *otx5*, *pde6a*, *rho*) using IGV software, we found only one paralog in *S. tileihornes* (Fig. S4), but not other eye-related genes. So, we first designed universal primers in the conserved regions of *otx5*. The sequencing results clearly show that *otx5* has double peaks and a 3-bp indel in the eye cDNAs of *S. angustiporus* and *S. anophthalmus*, while eye cDNA of *S. tileihornes* lacks the paralog with the indel (Fig. 5). In addition, PCR products with primer *otx5*-*F* and *otx5*-*R* were cloned into the pGM-T vector and sequenced (GenBank Accession Nos. MK983240-4). The transcriptome of *S. tileihornes* only contains *otx5a*, *S. angustiporus* and *S. anophthalmus* have both *otx5a* and *otx5b* (Figs. 5b and S5a). A single amino acid, Serine, is missing on the exon3 of *otx5b* caused by this 3-nt deletion in *S. angustiporus* and *S. anophthalmus* (Fig. S5b). Next, we designed paralog-specific primers for *crx* based on the SNP site that distinguishes *crxa* from *crxb* and *otx5* in the indel region that distinguishes *otx5a* from *otx5b* (Fig. S5a). The qRT-PCR results revealed no significant difference in the expression of *crxa* or *crxb* in *S. tileihornes* relative to their orthologs in the surface species *S. angustiporus* (Fig. 6a). We found that the expression of *otx5a* in *S. tileihornes* was comparable to that of *otx5a* in the surface species, but the expression of *otx5b* was missing in *S. tileihornes*. This result leads to the conclusion that the total expression of *otx5* (*otx5a* plus *otx5b*) in *S. tileihornes* decreased significantly relative to *S. angustiporus* (Fig. 6a). Using ISH, we noted that *otx5* mRNA was expressed in the neural retina of three species. However, its expression range and intensity were reduced in both cavefish *S. tileihornes* and *S. anophthalmus* (Fig. 6b). *Otx5* can cooperate with *crx* and neural retina-specific leucine zipper protein (*nrl*) to activate the expression of *rho* and other photoreceptor genes, as well as to orchestrate photoreceptor cell differentiation (Gamse et al. 2002; Reks et al. 2014). The reduction of *otx5* expression in *S. tileihornes* eyes may also contribute to the observed abnormality in rod cells. Experiments to test the hypothesis that *otx5b* plays a direct role in rod degeneration in cavefish require further investigation. The loss in *otx5b* expression in the eyes of *S. tileihornes* may have been caused by as yet unknown mutations in the *otx5b* promotor, which could lead to it being unresponsive to the upstream regulatory proteins of *otx5*, or by gene loss after the whole genome duplication.

## Electronic supplementary material

Below is the link to the electronic supplementary material.

Supplementary material 1 (DOCX 14 kb)

Supplementary material 2 (XLSX 4646 kb)

Supplementary material 3 (XLSX 2017 kb)

Supplementary material 4 (XLSX 17 kb)

Supplementary material 5 (DOCX 12 kb)

Supplementary material 6 (PDF 2020 kb)

## Data Availability

SRA accession numbers for RNA-seq are SRP150385 (*S. tileihornes*), SRR788094 (*S. angustiporus*) and SRR788095 (*S. anophthalmus*). The datasets supporting this article have been uploaded as part of the electronic supplementary material.
