# Supplementary material for: Eye Degeneration and Loss of otx5b Expression in the Cavefish Sinocyclocheilus tileihornes
Source: J Mol Evol. 2019 Jul 22;87(7):199–208. doi: 10.1007/s00239-019-09901-8 (PMC6711879; doi:10.1007/s00239-019-09901-8)
Supplement: Supplementary file 1 — Supplementary material 1 (DOCX 14 kb) [file 239_2019_9901_MOESM1_ESM.docx]

Eye degeneration and loss of *otx5b* expression in the cavefish *Sinocyclocheilus tileihornes*

Zushi Huang^1^, Tom Titus^2^, John H. Postlethwait^2§^, Fanwei Meng^1§^

1 Institute of Zoology, Chinese Academy of Sciences, Beijing 100101, China

2 Institute of Neuroscience, University of Oregon, Eugene, OR 97403, USA

§Author for correspondence:

Fanwei Meng

e-mail: [mengfw@ioz.ac.cn](mailto:mengfw@ioz.ac.cn)

John H. Postlethwait

e-mail: [jpostle@uoneuro.uoregon.edu](mailto:jpostle@uoneuro.uoregon.edu)

**Materials and Methods**

We reconstructed phylogenetic topologies using *otx5* and *crx* genes by maximum-likelihood (ML) and Bayesian inference (BI) methods. We used the HKY+G model chosen by the Bayesian information criteria using jModelTest 2.1.1(Posada 2008). We used PhyML 3.0 (Guindon and Gascuel 2003) to estimate the ML tree based on 1000 non-parametric bootstrap replicates. BI was performed using MrBayes 3.2.1 (Ronquist et al. 2012) with two independent runs including one cold and three heated chains. Markov chains were run for 10^7^ generations, and the initial 25% of trees were discarded as burn-in. We sampled 15,002 trees to estimate the majority-rule consensus tree and Bayesian posterior probabilities (BPP). Phylogenetic analysis was conducted twice using different random numbers.

**Reference**:

Guindon S, Gascuel O (2003) A simple, fast, and accurate algorithm to estimate large phylogenies by maximum likelihood. Syst Biol 52:696

Posada D (2008) jModelTest: phylogenetic model averaging. Mol Biol Evol 25:1253

Ronquist F, Teslenko M, van der Mark P, Ayres DL, Darling A, Hohna S, Larget B, Liu L, Suchard MA, Huelsenbeck JP (2012) MrBayes 3.2: efficient Bayesian phylogenetic inference and model choice across a large model space. Syst Biol 61:539
