# Supplementary material for: Eye Degeneration and Loss of otx5b Expression in the Cavefish Sinocyclocheilus tileihornes
Source: J Mol Evol. 2019 Jul 22;87(7):199–208. doi: 10.1007/s00239-019-09901-8 (PMC6711879; doi:10.1007/s00239-019-09901-8)
Supplement: Supplementary file 5 — Supplementary material 5 (DOCX 12 kb) [file 239_2019_9901_MOESM5_ESM.docx]

Figure S1. Cladogram of species referred to in this paper. (Adapted from Zhao and Zhang 2009, Meng et al 2013).

Figure S2. Conserved synteny analysis shows that the teleost genes called *crx* and *otx5* are ohnologs derived from the teleost genome duplication event, contrary to the zebrafish nomenclature conventions of making the zebrafish ohnologs use the same name and abbreviation as the mammalian gene except for italicizing all letters and using lower case followed by "a" or "b" to indicate that they are duplicate copies (see https://wiki.zfin.org/display/general/ZFIN+Zebrafish+Nomenclature+Conventions). The two zebrafish (*Danio rerio*) chromosomes Dre5 and Dre15 have substantial numbers of ohnologs derived from the TGD, as indicated by lines connecting genes on the representations of the two chromosomes.

Figure S3. Phylogenetic relationships of *otx5* and *crx* genes in *Sinocyclocheilus* and *Danio rerio*. These genes are co-orthologs of the human *CRX* gene that arose in the teleost genome duplication. Phylogenetic trees were reconstructed by maximum-likelihood (ML) and Bayesian inference (BI) methods. Bootstrap values > 70% and Bayesian posterior probabilities > 0.9 are shown on the tree. Abbreviations: *Sang*, *S. angustiporus*; *Sano*, *S. anophthalmus*; *Stil*, *S. tileihornes*; *Sans, S. anshuiensis*; *Sgra, S. grahami*; *Srhi*, *S. rhinocerous*; *Drer*, *Danio rerio*.

Figure S4. The mapping of short-read sequences of three species on the reference *otx5* gene was visualized by IGV software. Red arrows represent single nucleotide polymorphisms (SNPs) between the co-paralogs *otx5a* and *otx5b,* which derive from the carp genome duplication event.

Figure S5. (a) Sequence alignments of *otx5* among three species. The yellow line indicates the universal primers in the conserved regions of the *otx5*. The paralogue-specific primers for *otx5a* and *otx5b* in the gap region are indicated by red and blue lines, respectively. (b) A single amino acid is missing on the exon3 of *otx5b* caused by this 3-nt deletion. *Sang*, *S. angustiporus*; *Sano*, *S. anophthalmus*; *Stil*, *S. tileihornes*. A, Alanine; K, Lysine; S, Serine; T, Threonine; W, Tryptophan.
