# Supplementary material for: Eye Degeneration and Loss of otx5b Expression in the Cavefish Sinocyclocheilus tileihornes
Source: J Mol Evol. 2019 Jul 22;87(7):199–208. doi: 10.1007/s00239-019-09901-8 (PMC6711879; doi:10.1007/s00239-019-09901-8)
Supplement: Supplementary file 6 — Supplementary material 6 (PDF 2020 kb) [file 239_2019_9901_MOESM6_ESM.pdf]

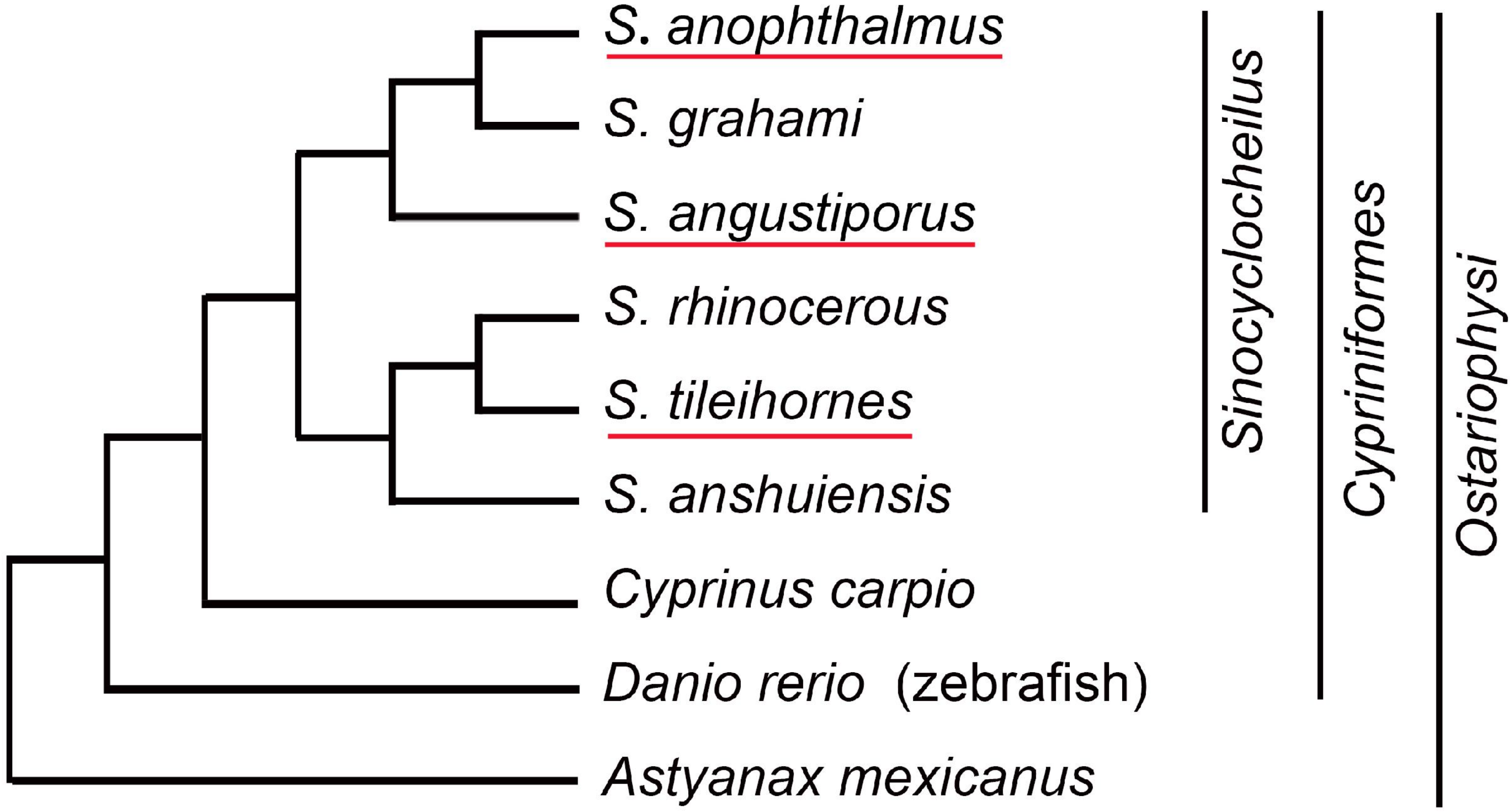
S. anophthalmus
S. grahami
S. angustiporus
S. rhinocerous
S. tileihornes
S. anshuiensis
Cyprinus carpio
Danio rerio (zebrafish)
Astyanax mexicanus
Sinocyclocheilus
Cypriniformes
Ostariophysi

Fig. S1

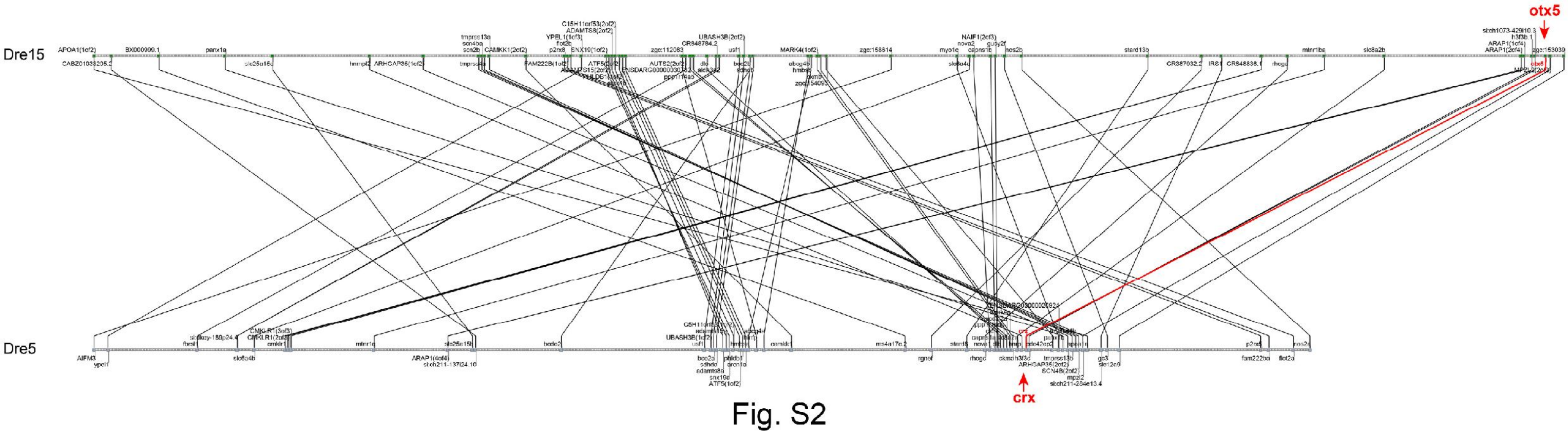


Fig. S2

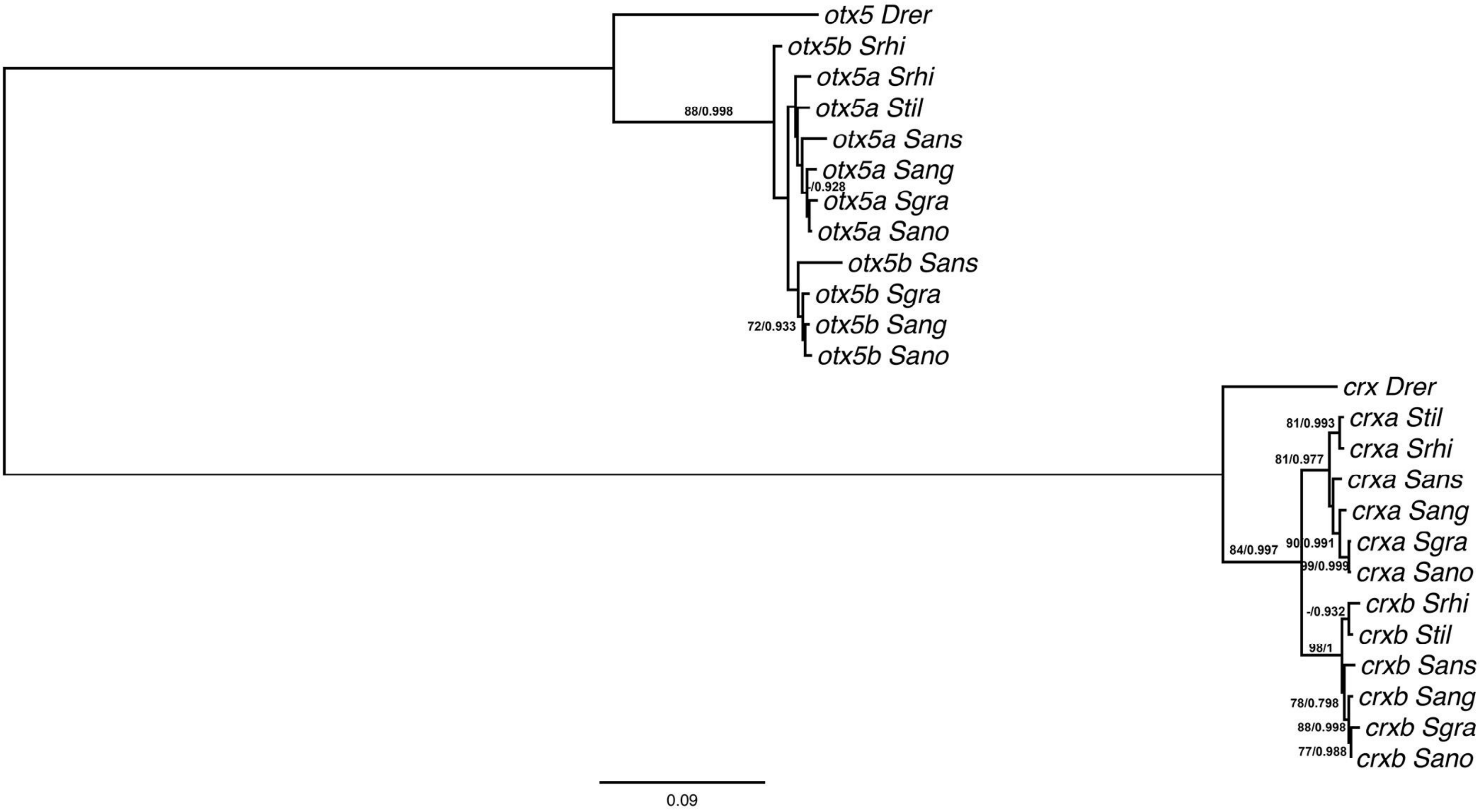
otx5 Drer
otx5b Srhi
otx5a Srhi
otx5a Stil
otx5a Sans
otx5a Sang
otx5a Sgra
otx5a Sano
otx5b Sans
otx5b Sgra
otx5b Sang
otx5b Sano
88/0.998
-/0.928
72/0.933
crx Drer
crxa Stil
crxa Srhi
crxa Sans
crxa Sang
crxa Sgra
crxa Sano
crxb Srhi
crxb Stil
crxb Sans
crxb Sang
crxb Sgra
crxb Sano
81/0.993
81/0.977
84/0.997
90/0.991
99/0.999
-/0.932
98/1
78/0.798
88/0.998
77/0.988
0.09

Fig. S3

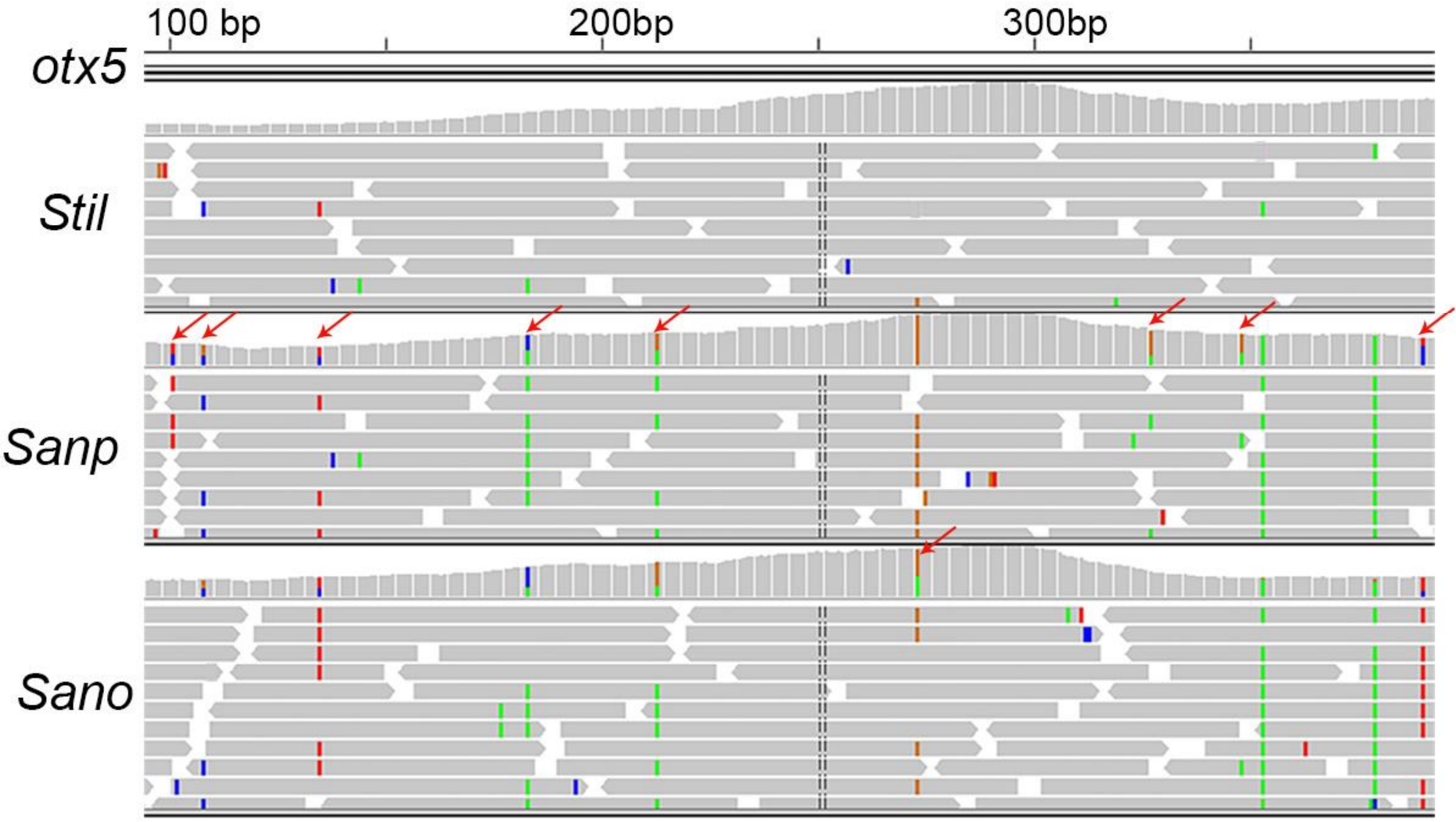


Fig. S4

A

```
otx5b-Sano   ATGATGTCCTACATGAAGCAGCCTCATTACTCGGTGAACGGCTTGACTCTGTCCGGTACC   60
otx5b-Sanp   ATGATGTCCTACATGAAGCAGCCTCATTACTCGGTGAACGGCTTGACTCTGTCCGGTACC   60
otx5a-Stil   ATGATGTCCTACATGAAGCAGCCTCATTACTCGGTGAACGGCTTGACTCTGTCCGGTACC   60
otx5a-Sano   ATGATGTCCTACATGAAGCAGCCTCATTACTCGGTGAACGGCTTGACTCTGTCCGGTACC   60
otx5a-Sanp   ATGATGTCCTACATGAAGCAGCCTCATTACTCGGTGAACGGCTTGACTCTGTCCGGTACC   60
             ************************************************************

otx5b-Sano   GGGATGGATCTCCTGCACTCCGCTGTCGGTTACCCCAACACTCCGCGCAAGCAGCGGCGC   120
otx5b-Sanp   GGGATGGATCTCCTGCACTCCGCTGTCGGTTACCCCAACACTCCGCGCAAGCAGCGGCGC   120
otx5a-Stil   GGGATGGATCTCCTGCACTCCGCTGTCGGTTACCCCAACACTCCGCGGAAGCAGCGGCGC   120
otx5a-Sano   GGGATGGATCTCCTGCACTCCGCTGTCGGTTACCCCAACACTCCGCGGAAGCAGCGGCGC   120
otx5a-Sanp   GGGATGGATCTCCTGCACTCCGCTGTCGGTTACCCCAACATTCCGCGGAAGCAGCGGCGC   120
             **************************************** ****** ************

otx5b-Sano   GAGAGAACCACCTTCACGCGCGCGCAGCTCGACATCCTCGAGTCACTGTTCGCCAAGACA   180
otx5b-Sanp   GAGAGAACCACCTTCACGCGCGCGCAGCTCGACATCCTCGAGTCACTGTTCGCCAAGACA   180
otx5a-Stil   GAGAGAACCACCTTTACGCGCGCGCAGCTCGACATCCTCGAGTCACTGTTCGCCAAGACA   180
otx5a-Sano   GAGAGAACCACCTTTACGCGCGCGCAGCTCGACATCCTCGAGTCACTGTTCGCCAAGACA   180
otx5a-Sanp   GAGAGAACCACCTTTACGCGCGCGCAGCTCGACATCCTCGAGTCACTGTTCGCCAAGACA   180
             ************** *********************************************

otx5b-Sano   CGATACCCAGACATCTTCATGAGAGAGGACGTAGCTCTCAAGATCAACCTGCCCGAGTCC   240
otx5b-Sanp   CGATACCCAGACATCTTCATGAGAGAGGACGTAGCTCTCAAGATCAACCTGCCCGAGTCC   240
otx5a-Stil   CGCTACCCAGACATCTTCATGAGAGAGGACGTGGCTCTCAAGATCAACCTGCCCGAGTCC   240
otx5a-Sano   CGCTACCCAGACATCTTCATGAGAGAGGACGTGGCTCTCAAGATCAACCTGCCCGAGTCC   240
otx5a-Sanp   CGCTACCCAGACATCTTCATGAGAGAGGACGTGGCTCTCAAGATCAACCTGCCCGAGTCC   240
             ** ***************************** ***************************

otx5b-Sano   AGAGTCCAGGTGTGGTTTAAGAACCGTCGTGCAAAGTGCCGTCAACAGCAGCAGCAACAG   300
otx5b-Sanp   AGAGTCCAGGTGTGGTTTAAGAACCGTCGTGCGAAGTGCCGTCAACAGCAGCAGCAACAG   300
otx5a-Stil   AGAGTCCAGGTGTGGTTTAAGAACCGTCGTGCGAAGTGCCGTCAACAGCAGCAGCAACAG   300
otx5a-Sano   AGAGTCCAGGTGTGGTTTAAGAACCGTCGTGCAAAGTGCCGTCAACAGCAGCAGCAACAG   300
otx5a-Sanp   AGAGTCCAGGTGTGGTTTAAGAACCGTCGTGCGAAGTGCCGTCAACAGCAGCAGCAACAG   300
             ******************************** ***************************
                               otx5-F

otx5b-Sano   ACCAGCGGCCAGACCAAACCCAGACCGCCGAAGAAGAAGTCGTCTCCGGCCCAAGAGCCC   360
otx5b-Sanp   ACCAGCGGCCAGACCAAACCCAGACCGCCGAAGAAGAAGTCGTCTCCGGCCCAAGAGCCC   360
otx5a-Stil   ACCAGCGGCCAGACCAAACCCAGACCACCGAAGAAGTCGTCGTCTCCGGCCCAAGAGCCC   360
otx5a-Sano   ACCAGCGGCCAGACCAAACCCAGACCGCCGAAGAAGAAGTCGTCTCCGGCCCAAGAGCCC   360
otx5a-Sanp   ACCAGCGGCCAGACCAAACCCAGACCACCGAAGAAGAAGTCGTCTCCGGCCCAAGAGCCC   360
             ************************** *********  **********************

otx5b-Sano   AGCGTCAGCGAGCCCAGCACCAGCACCAATGGCCCCTACAGCCCTCCGCCTCCTCCGGCC   420
otx5b-Sanp   AGCGTCAGCGAGCCCAGCACCAGCACCAATGGCCCCTACAGCCCTCCGCCTCCTCCGGCC   420
otx5a-Stil   AGCGTCAGCGAGCCCAGCACCAGCACCAACGGCCCCTACAGCCCTCCGCCTCCTCCGGCC   420
otx5a-Sano   AGCGTCAGCGAGCCCAGCACCAGCACCAATGGCCCCTACAGCCCTCCGCCTCCTCCGGCC   420
otx5a-Sanp   AGCGTCAGCGAGCCCAGCACCAGCACCAATGGCCCCTACAGCCCTCCGCCTCCTCCGGCC   420
             ***************************** ******************************

otx5b-Sano   CCGAGCTCCAGCTCCACCAGCGCCACCGTGTCCATCTGGAGCCCGGCCTCCATCTCTCCG   480
otx5b-Sanp   CCGAGCTCCAGCTCCACCAGCGCCACCGTGTCCATCTGGAGCCCGGCCTCCATCTCTCCG   480
otx5a-Stil   CCGAGCTCCAGCTCCACCAGCGCCACCGTGTCCATCTGGAGCCCGGCCTCCATCTCTCCG   480
otx5a-Sano   CCGAGCTCCAGCTCCACCAGCGCCACCGTGTCCATCTGGAGCCCGGCCTCCATCTCTCCG   480
otx5a-Sanp   CCGAGCTCCAGCTCCACCAGCGCCACCGTGTCCATCTGGAGCCCGGCCTCCATCTCTCCG   480
             ************************************************************

otx5b-Sano   CTTCAGGACCCACTGTCGGTCTCCAGCACGCCCTGCCTGCAGCGCTCCAGCGCCTACCCC   540
otx5b-Sanp   CTTCAGGACCCGCTGTCGGTCTCCAGCACGCCCTGCCTGCAGCGCTCCAGCGCCTACCCC   540
otx5a-Stil   CTTCAGGACCCGCTGTCGGTCTCCAGCACGCCCTGCCTGCAGCGCTCCAGCGCCTACCCC   540
otx5a-Sano   CTTCAGGACCCGCTGTCGGTCTCCAGCACGCCCTGCCTGCAGCGCTCCAGCGCCTACCCC   540
otx5a-Sanp   CTTCAGGACCCGCTGTCGGTCTCCAGCACGCCCTGCCTGCAGCGCTCCAGCGCCTACCCC   540
             *********** ************************************************

otx5b-Sano   ATGACCTACACCCAGGCCCCGGCCTACGGGCAGAGCTACGCCGCCTCCTCGTCCTACTTC   600
otx5b-Sanp   ATGACCTACACCCAGGCCCCGGCCTACGGGCAGAGCTACGCTGCCTCCTCGTCCTACTTC   600
otx5a-Stil   ATGACCTACAGCCAGGCCCCGGCCTACGGGCAGAGCTACGCCGCCTCCTCGTCCTACTTC   600
otx5a-Sano   ATGACCTACACCCAGGCCCCGGCCTACGGGCAGAGCTACGCCGCCTCCTCGTCCTACTTC   600
otx5a-Sanp   ATGACCTACACCCAGGCCCCGGCCTACGGGCAGAGCTACGCCGCCTCCTCGTCCTACTTC   600
             ********** ****************************** ******************
                                                         otx5ab-F

otx5b-Sano   ACCGGGCTGGACTGCAGCTCGTATCTGTCCCCCATGCACCCGCAGCTGTCGGCCACAGGG   660
otx5b-Sanp   ACCGGGCTGGACTGCAGCTCATATCTGTCCCCCATGCACCCGCAGCTGTCGGCCACAGGG   660
otx5a-Stil   ACCGGGCTGGACTGCAGCTCATATCTGTCCCCCATGCACCCGCAGCTGTCGGCCACAGGG   660
otx5a-Sano   ACCGGGCTGGACTGCAGCTCGTATCTGTCCCCCATGCACCCGCAGCTGTCGGCCACAGGG   660
otx5a-Sanp   ACCGGGCTGGACTGCAGCTCGTATCTGTCCCCCATGCACCCGCAGCTGTCGGCCACAGGG   660
             ******************** ***************************************

otx5b-Sano   GGCGCTCTCAGCCCCATGGGTGCAGCGCTCAGCCAGTCGCCCGCCTCGCTCTCGTCGCAG   720
otx5b-Sanp   GGCGCTCTCAGCCCCATGGGTGCAGCGCTCAGCCAGTCGCCCGCCTCGCTCTCGTCGCAG   720
otx5a-Stil   GGCGCTCTCAGCCCCATGGGTGCAGCGCTCAGCCAGTCCCCCGCCTCGTTCTCGTCGCAG   720
otx5a-Sano   GGCGCTCTCAGCCCCATGGGTGCAGCGCTCAGCCAGTCCCCCGCCTCGTTCTCGTCGCAG   720
otx5a-Sanp   GGCGCTCTCAGCCCCATGGGTGCAGCGCTCAGCCAGTCCCCCGCCTCGTTCTCGTCGCAG   720
             ************************************** ********* ***********

otx5b-Sano   GGCTACACGGCCGCTTCGCTGGGCTTCGGCGCCATCGACTGCTTAGACTACAAAGACCAA   780
otx5b-Sanp   GGCTACACGGCCGCTTCGCTGGGCTTCGGCGCCGTCGACTGCTTAGACTACAAAGACCAA   780
otx5a-Stil   GGCTACACCGCCGCGTCACTGGGCTTCGGCGCCGTCGACTGCTTAGACTACAAAGACCAA   780
otx5a-Sano   GGCTACACCGCCGCGTCGCTGGGCTTCGGTGCCGTCGACTGCTTAGACTACAAAGACCAA   780
otx5a-Sanp   GGCTACACGGCCGCGTCGCTGGGCTTCGGCGCCGTCGACTGCTTAGACTACAAAGACCAA   780
             ******** ***** ** *********** *** **************************

otx5b-Sano   ACGGC---CTGGAAGCTCAACTTCAACGCTGCGGACTGCCTGGACTACAAGGACCAGAAC   837
otx5b-Sanp   ACGGC---CTGGAAGCTCAACTTCAACGCTGCGGACTGCCTGGACTACAAGGACCAGAAC   837
otx5a-Stil   ACGGCGTCCTGGAAGCTCAACTTCAACGCTGCGGACTGTCTGGACTACAAGGACCAGAAC   840
otx5a-Sano   ACGGCGTCCTGGAAGCTGAACTTCAACGCTGCGGACTGTCTGGACTACAAAGACCAGAAC   840
otx5a-Sanp   ACGGCGTCCTGGAAGCTGAACTTCAACGCTGCGGATTGTCTGGACTACAAAGACCAGAAC   840
             *****   ********* ***************** ** *********** *********
             otx5a-R and otx5b-R

otx5b-Sano   TCCTGGAAGTTCCAGGTCCTGTAG   861
otx5b-Sanp   TCCTGGAAGTTCCAGGTCCTGTAG   861
otx5a-Stil   TCCTGGAAGTTCCAGGTCCTGTAG   864
otx5a-Sano   TCCTGGAAGTTCCAGGTCCTGTAG   864
otx5a-Sanp   TCCTGGAAGTTCCAGGTCCTGTAG   864
             ************************
               otx5-R
```

B

```
otx5b-mRNA     ACGGC---CTGGAAG
otx5b-protein   T  A     W  K
                |  |     |  |
otx5a-protein   T  A  S  W  K
otx5a-mRNA     ACGGCGTCCTGGAAG
```

Fig. S5
